# Sustained efficacy of chimeric antigen receptor T-cell therapy in central nervous system lymphoma: a systematic review and meta-analysis of individual data

**DOI:** 10.3389/fphar.2023.1331844

**Published:** 2024-01-24

**Authors:** Jing Zhou, Zhenhao Wang, Hanyu Wang, Yang Cao, Gaoxiang Wang

**Affiliations:** Department of Hematology, Tongji Hospital, Tongji Medical College, Huazhong University of Science and Technology, Wuhan, China

**Keywords:** CAR T-cell therapy, CNSL, systematic review, meta-analysis, immunothearpy

## Abstract

**Background:** Central nervous system lymphoma (CNSL) is considered an aggressive lymphoma with a poor prognosis. Studies investigating CNSL have shown that chimeric antigen receptor (CAR) T-cell therapy has demonstrated an effective response in limited sample sizes. Therefore, we conducted this systematic review and meta-analysis to clarify the sustained efficacy and factors associated with the sustained efficacy of CAR T-cell therapy in the treatment of CNSL.

**Methods:** We searched studies from PubMed, Embase, Medline, and the Cochrane Center Register of Controlled Trials up to July 2023. Studies that included individual data on the duration of response (DoR) after receiving CAR T-cell therapy were enrolled. Pooled response rates were calculated using fixed-effects or random-effects models. Subgroup analysis was performed to analyze the heterogeneity, and a Cox regression model was performed to identify the factors associated with sustained efficacy.

**Results:** In total, 12 studies including 69 patients were identified and included in this meta-analysis. The pooled relapse rate was 45% [95% CI 35, 56]. Subgroup analyses of relapse rates revealed that CAR T-cells using the CD28/4-1BB domain (CD28/4-1BB vs. CD28 vs. 4-1BB, *p* = 0.0151), parenchymal or leptomeningeal involvement (parenchymal or leptomeningeal vs. both parenchymal and leptomeningeal, *p* < 0.0001), and combined treatment with CAR T-cell therapy [Autologous stem cell transplantation (ASCT) plus CAR T-cell therapy vs. CAR T cells with maintenance therapy vs. CAR T-cell therapy alone, *p* = 0.003] were associated with lower relapse rates in patients. Time-to-event endpoints were assessed using reconstructed individual patient survival data to explore key modulators of DoR. Partial response status at CAR-T infusion and the use of ASCT plus CAR T-cell therapy were associated with longer DoR at the multivariate level, with hazard ratios of 0.25 and 0.26, respectively.

**Conclusion:** CAR T-cell therapy shows promising and sustained efficacy in CNSL patients. However, further prospective large-scale studies are needed to assess these effect modifiers to optimize patient selection and improve the sustained efficacy of CAR T-cell therapy in the treatment of CNSL.

**Systematic review registration:** https://clinicaltrials.gov/, identifier PROSPERO CRD42023451856.

## Introduction

Central nervous system lymphoma (CNSL) is a rare type of non-Hodgkin’s lymphoma (NHL), that is categorized into two main types: primary CNSL (PCNSL) and secondary CNSL (SCNSL). PCNSL is a high-grade extranodal non-Hodgkin lymphoma characterized by the abnormal proliferation of malignant lymphocytes within the central nervous system, including the brain, leptomeninges, eye, and spinal cord. SCNSL differs from PCNSL, defined as secondary involvement of the neuroaxis due to systemic disease (Villano et al., 2011) (Grommes and DeAngelis, 2017). Although the therapeutic effect has been improved by the use of high-dose chemotherapy followed by autologous hematopoietic stem cell transplantation, the prognosis is still not optimistic, with a high tendency for recurrence (Dahiya et al., 2013; El-Galaly et al., 2018). Therefore, finding novel effective therapies to prolong the duration of response (DoR) in CNSL patients is an urgent need.

Chimeric antigen receptor (CAR) T-cell therapy has demonstrated prominent therapeutic efficacy in hematologic malignancies, such as B-acute lymphoblastic leukemia, diffuse large B-cell lymphoma (DLBCL), multiple myeloma, mantle cell lymphoma and follicular lymphoma (Davila et al., 2014; Abramson et al., 2017; Ghione et al., 2021; Haslauer et al., 2021). As of December 2023, the following four CAR-T cell therapies have been approved: KYMRIAH (tisagenlecleucel) for adult patients with relapsed or refractory (r/r) follicular lymphoma (FL) (Fowler et al., 2022), YESCARTA (axicabtagene ciloleucel) for adult patients with (r/r) large B-cell lymphoma (LBCL) (Locke et al., 2019), Tecartus (brexucabtagene autoleucel) for adult patients with (r/r) mantle cell lymphoma (MCL) (Wang et al., 2023), and BREYANZI (lisocabtagene maraleucel) for adult patients with r/r LBCL (Abramson et al., 2020). This revolutionary form of immunotherapy is achieved through the modification of immune cells, conferring to them the capability to identify and eliminate tumor cells by targeting distinct surface proteins. However, notwithstanding the remarkable clinical achievements, a primary challenge in CAR T-cell therapy is the ambiguity about its durability. Additionally, patients with lymphoma involving the CNS were often excluded from pivotal studies due to concerns regarding neurotoxicity associated with CAR T-cell therapy. In 2017, Abramson et al. first demonstrated the efficacy of CD19-specific CAR T-cell in secondary CNSL, suggesting the feasibility of CAR T-cell therapy in CNSL patients (Abramson et al., 2017). Following this, several studies have endorsed the efficacy of CAR T-cell therapy in CNSL, whether it is PCNSL or SCNSL, with controllable adverse events.

A few previous studies assessed the efficacy of CAR T-cell therapy in CNSL patients, but they did not evaluate the duration response to this therapy (Cook et al., 2023; Zinzi et al., 2023). Herein, we conducted a systematic review and meta-analysis to evaluate the DoR of CAR T-cell therapy in CNSL patients and the associated factors.

## Materials and methods

### Search strategy

Two investigators independently searched published studies on CAR T-cell therapy in CNSL patients before July 2023 in PubMed, Embase, Medline, and the Cochrane Center Register of Controlled Trials. The following MeSH and Entry terms were used to construct the search strategy: (Receptors, Chimeric Antigen OR Antigen Receptors, Chimeric OR Chimeric Antigen Receptors OR Chimeric Antigen Receptor OR CAR OR Antigen Receptor, Chimeric OR Receptor, Chimeric Antigen OR Chimeric T-Cell Receptors OR Chimeric T Cell Receptors OR Receptors, Chimeric T-Cell OR T-Cell Receptors, Chimeric OR Artificial T-Cell Receptors OR Artificial T Cell Receptors OR Receptors, Artificial T-Cell OR T-Cell Receptors, Artificial OR Chimeric T-Cell Receptor OR Chimeric T Cell Receptor OR Receptor, Chimeric T-Cell OR T-Cell Receptor, Chimeric OR Chimeric Immunoreceptors) AND (Central Nervous System Neoplasms Neoplasms OR Central Nervous System OR Central Nervous System Tumor OR Tumors, Central Nervous System OR Central Nervous System Neoplasm OR CNS Neoplasm OR CNS Neoplasms OR Neoplasm, CNS OR Neoplasms, CNS OR Central Nervous System Tumors OR Central Nervous System Neoplasms, Primary OR Primary Central Nervous System Neoplasm OR Primary Central Nervous System Neoplasms OR brain lymphoma OR central nervous system lymphoma OR CNS lymphoma OR CNSL OR CNSLs). Additionally, we boosted our literature search through a manual search of the reference lists of eligible articles. The Preferred Reporting Items for Systematic Reviews and Meta-Analyses (PRISMA) statement eligibility criteria were followed in this study. This meta-analysis has been registered in the International Prospective Register of Systematic Reviews (PROSPERO) (CRD42023451856).

### Eligibility criteria

We included reports containing individual data on the DoR after receiving CAR T-cell therapy in adult patients diagnosed as CNSL from clinical trials, prospective and retrospective cohort studies, and conference abstracts. We excluded case reports, reviews, comments, and other literature with unavailable study data. Studies published in English were included regardless of the number of participants.

Two researchers independently assessed the records by reading the titles and abstracts. The full texts were obtained for all reports that seemed to meet the inclusion criteria or in cases of ambiguity. Subsequently, the remaining records were evaluated independently by two researchers to determine their compliance with the inclusion criteria. If there was a disagreement, it was resolved by adding a third researcher.

### Quality assessment

The methodological index for non-randomized studies (MINORS) was used to evaluate the prospective non-randomized studies (single-arm studies) (Slim et al., 2003). The JBI Critical Appraisal Checklist for Case Series was used to evaluate the retrospective studies without a comparison group (Moola et al., 2015).

### Data extraction

The main outcome of the study was DoR after CAR T-cell infusion. DOR was defined as the time from best overall response (BOR), the first documented complete or partial response, to disease progression. In addition, data from each study were extracted: first author, year, sample size, median age, sex, etiology (primary or secondary), sites of central nervous system (CNS) involvement, status at CAR T-cell infusion, combination therapy along with CAR T-cell therapy, number of previous therapies, prior autologous stem cell transplantation (ASCT), and disease status (systemic + CNS, isolated CNS).

### Statistical analysis

Data analysis was conducted in R (version 4.3.0) using R Studio (version 2023.3.1.446). Pooled rates were calculated employing either a random effects model or a fixed effect model with double arcsine transformation. The 95% confidence interval (CI) was utilized to denote the effect size of the combined outcomes, accounting for both the upper and lower limits. Heterogeneity assessment between studies involved Cochran’s Q test and I^2^ statistics. Pooled results characterized by low heterogeneity (I^2^ ≤ 50%) were analyzed using a fixed-effects model, while a random-effects model was employed for cases of higher heterogeneity. Sensitivity analysis involved the sequential exclusion of each study from the pooled results in instances of high heterogeneity. Additionally, potential publication bias in the included studies was investigated using inspection of the funnel plots, and Begg’s and Egger’s tests.

To estimate the time-to-event endpoint (DoR), OriginPro 2021 (Originlab, 2021) was used to extract data through manual selection in order to retrieve the coordinates of the target points (BoR point and corresponding relapse point). Univariate and multivariate Cox regression analyses were performed to explore the impact of variables on the DoR by using RStudio software’s survminer package. The distribution of DoR was estimated using the Kaplan–Meier method with GraphPad Prism software (8.0.1).

## Result

**FIGURE 1:**
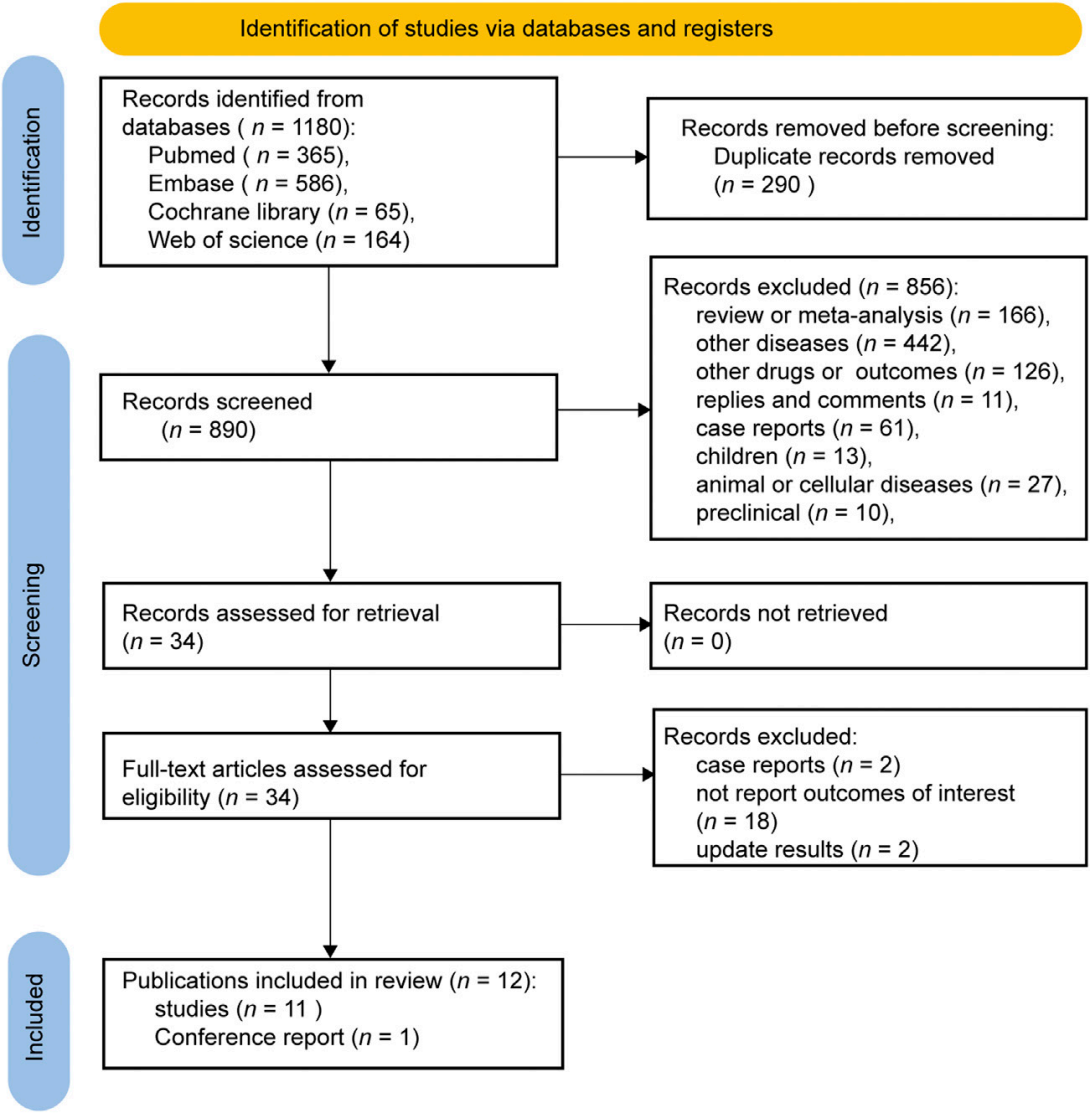
Flow diagram of the study selection process.

Initially, a total of 1,180 relevant reports were identified, of which 12 studies were included according to our eligibility criteria after de-duplication and screening the title, abstract, and full text (Figure 1). The included studies were published between 2019 and 2023 and deemed relatively high quality (Table 1). The meta-analysis analyzed the sustained efficacy of CAR T-cell therapy for a total of 69 patients with CNSL, who achieved an objective response [complete response (CR) or partial response (PR)], across 12 cohort studies (Frigault et al., 2019; Li et al., 2020; Ahmed et al., 2021; Ghafouri et al., 2021; Roddie et al., 2021; Siddiqi et al., 2021; Wu et al., 2021; Frigault et al., 2022; Liu et al., 2022; Xue et al., 2022; Zhang et al., 2022; Lacan et al., 2023). These studies encompassed 27 PCNSL and 42 SCNSL, with sample sizes ranging from 2 to 14. Patients underwent CAR T-cell monotherapy in six studies (Frigault et al., 2019; Ahmed et al., 2021; Ghafouri et al., 2021; Roddie et al., 2021; Frigault et al., 2022; Zhang et al., 2022) and ASCT combined with CAR T-cell therapy in one study (Wu et al., 2021), while diverse treatments containing CAR T-cell therapy were employed in the remaining five studies (Li et al., 2020; Siddiqi et al., 2021; Liu et al., 2022; Xue et al., 2022; Lacan et al., 2023). For co-stimulation domains, two studies used CD28 (Ghafouri et al., 2021; Siddiqi et al., 2021), six studies used 4-1BB (Frigault et al., 2019; Roddie et al., 2021; Frigault et al., 2022; Liu et al., 2022; Xue et al., 2022; Zhang et al., 2022), two studies contained both CD28 and 4-1BB (Ahmed et al., 2021; Lacan et al., 2023), and two studies used CD28/4-1BB (Li et al., 2020; Wu et al., 2021). The baseline clinical characteristics of the patients are summarized in Table 2.

**TABLE 1:** The characteristics of included studies.

First Author, year	Study	Country	Design	Study period	MINORS score	JBI score
Frigault, 2019	tisagenlecleucel	America	retrospective	8/2018–3/2019	—	High
Li, 2020	third-generation CAR (CAR19/22) T-cell	China	prospective	NA	13	—
Siddiqi, 2021	CD19 CAR-T cells	America	prospective	as of 10/2020	14	—
Wu, 2021	ASCT + CD19/22 CAR-T cells	China	prospective	1/2019–2/2021	13	—
Liu, 2021	CD19 or CD 22 CAR-T cells with or without ASCT	China	prospective	11/2018–4/2021	15	—
Frigaul, 2021	Tisagenlecleucel	America	prospective	12/2019–11/2021	13	—
Zhang, 2022	CD19, CD20 or CD22 CAR-T cells	China	retrospective	7/2017–8/2021	—	High
Xue, 2022	CD19/CD20/CD22 CAR T-cell therapy with or without ASCT	China	retrospective	10/2018–10/2020	—	High
Ahmed, 2021	Tisagenlecleucel or Axicabtagene	America	retrospective	NA	—	High
Ghafouri, 2021	Axicabtagene	America	retrospective	10/2017–1/2020	—	High
Lacan,2023	Tisagenlecleucel or Axicabtagene	France	retrospective	1/2020–1/2022	—	High
Roddie, 2022	AUTO1	America	prospective	as of 2/2022	12	—

NA: Not available

**TABLE 2:** Baseline clinical characteristics of patients in the studies included in the systematic review.

First Author, year	Number of enrolled patients	Median age (year)	Sex male/female	Median n.of prior therapies (range)	Prior ASCT(yes/no)	Primary/Secondary CNS involvement	Disease status (systemic + CNS/Isolated CNS)	Types of CNS disease (parenchymal or leptomeningeal involvement/parenchymal and leptomeningeal involvement	Co-stimulation domain	Therapy	Status at CAR-T infusion	BOR	Patients’ status after BOR
Frigault, 2019	2	71.5 (64–79)	0/2	4.5 (4–5)	1/0	2 SCNSL	1/1	2/0	4-1BB	CAR-T alone	1SD 1PD	2 CR	1CR 1PD
Li, 2020	2	43.5 (38–49)	1/1	3 (2–4)	0/2	1 PCNSL; 1 SCNSL	1/1	2/0	CD28 and 4-1BB	CAR-T alone (n = 1) ASCT + CAR-T (n = 1)	2PD	1CR 1PR	1CR 1PD
Siddiqi, 2021	3	49 (42–53)	0/3	3 (2–5)	0/3	3 PCNSL	NA	NA	CD28	CAR-T alone (n = 2) CAR-T with maintain therapy (n = 1)	NA	3CR	2CR 1PD
Wu, 2021	8	41 (23–65)	5/3	3.5 (2–5)	0/8	2 PCNSL; 6 SCNSL	1/8	8/0	CD28 and 4-1BB	ASCT + CAR-T	1SD 5PD 2PR	6CR 2PR	6CR 1PR 1PD
Liu, 2021	7	48 (33–66)	3/4	5 (2–8)	NA	1 PCNSL; 6 SCNSL	2/4	7/0	4-1BB	CAR-T alone (n = 3) ASCT + CART(n = 2) CAR-T with maintain therapy (n = 2)	1SD 6PD	3CR 4PR	3CR 1PR 3PD
Frigaul 2021	7	NA	NA	4 (2–6)	2/5	7 PCNSL	NA	7/0	4-1BB	CAR-T alone	7SD	1CR 6PR	3CR 4PD
Zhang, 2022	2	NA	NA	NA	NA	2 SCNSL	1/NA	NA	4-1BB	CAR-T alone	2PR	2CR	2 PD
Xue, 2022	14	40.5 (19–66)	6/8	4 (2–11)	3/11	1 PCNSL; 13 SCNSL	4/8	11/3	4-1BB	CAR-T alone (n = 8) ASCT + CAR-T (n = 6)	8PD 6PR	7CR 7PR	6CR 8PD
Ahmed, 2021	5	50 (39–72)	2/3	4 (2–4)	1/4	5 SCNSL	2/3	5/0	CD28 (n = 2); 41-BB (n = 3)	CAR-T alone	4PD 1PR	5CR	3CR 2PD
Ghafouri, 2021	3	55 (28–76)	NA	3 (1–3)	0/3	3 SCNSL	2/1	1/2	CD28	CAR-T alone	NA	3CR	1CR 2PD
Lacan, 2023	13	69 (44–75)	4/9	3 (2–5)	12/1	9 PCNSL; 4 SCNSL	NA	2/NA	CD28 (n = 2); 41-BB (n = 11)	CAR-T alone (n = 12) CAR-T with maintain therapy (n = 1)	8PD 5PR	6CR 7PR	5CR 2PR 6PD
Roddie, 2022	3	NA	NA	NA	NA	3 PCNSL	NA	NA	4-1BB	CAR-T alone	NA	2CR 1PR	2CR 1PD

ASCT: autologous stem cell transplantation; CNS: central nervous system; CAR: chimeric antigen receptor; BOR: best overall response; SCNSL: secondary central nervous system lymphoma; PCNSL: primary central nervous system lymphoma; PD: progressive disease; SD: stable disease; PR: partial remission; CR: complete response; NA: not available.

**FIGURE 2:**
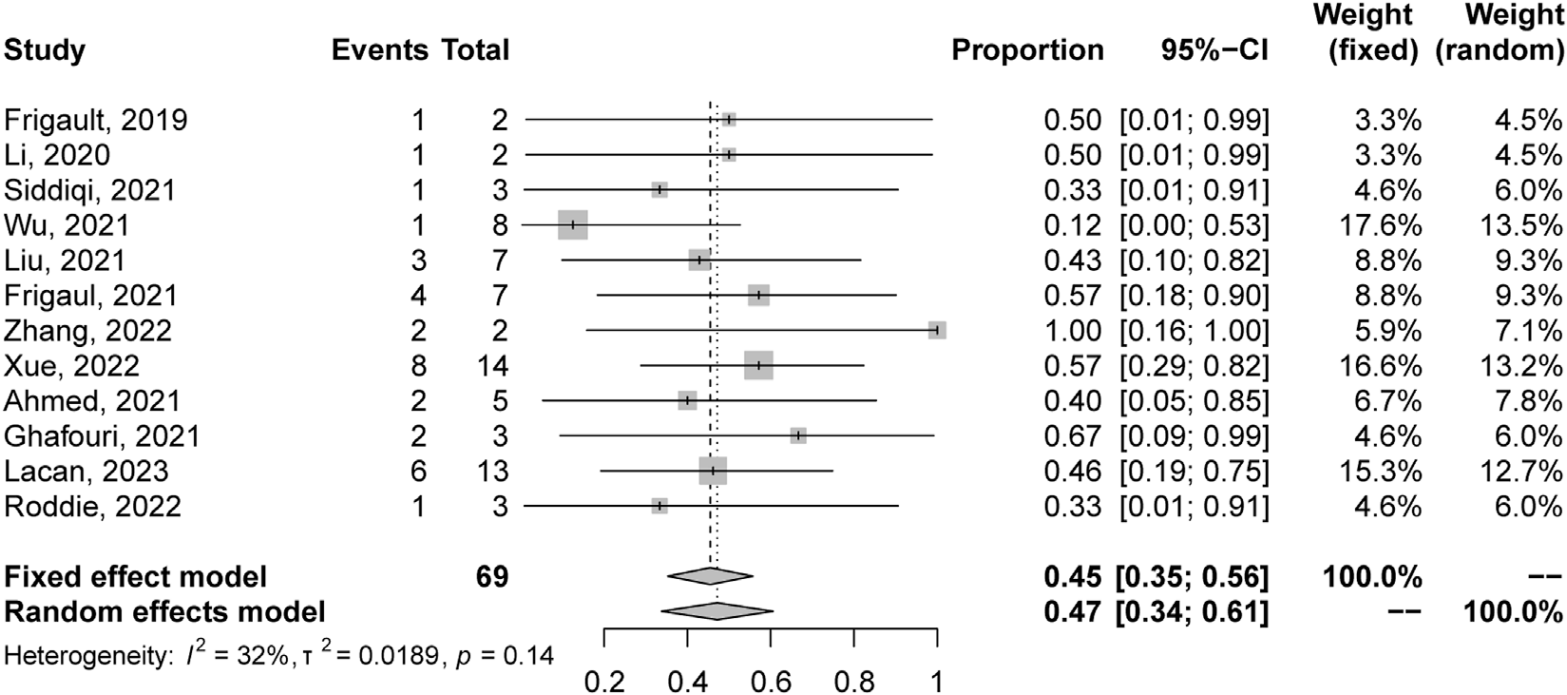
Forest plot of relapse rates and confidence intervals in CNSL patients.

**FIGURE 3:**
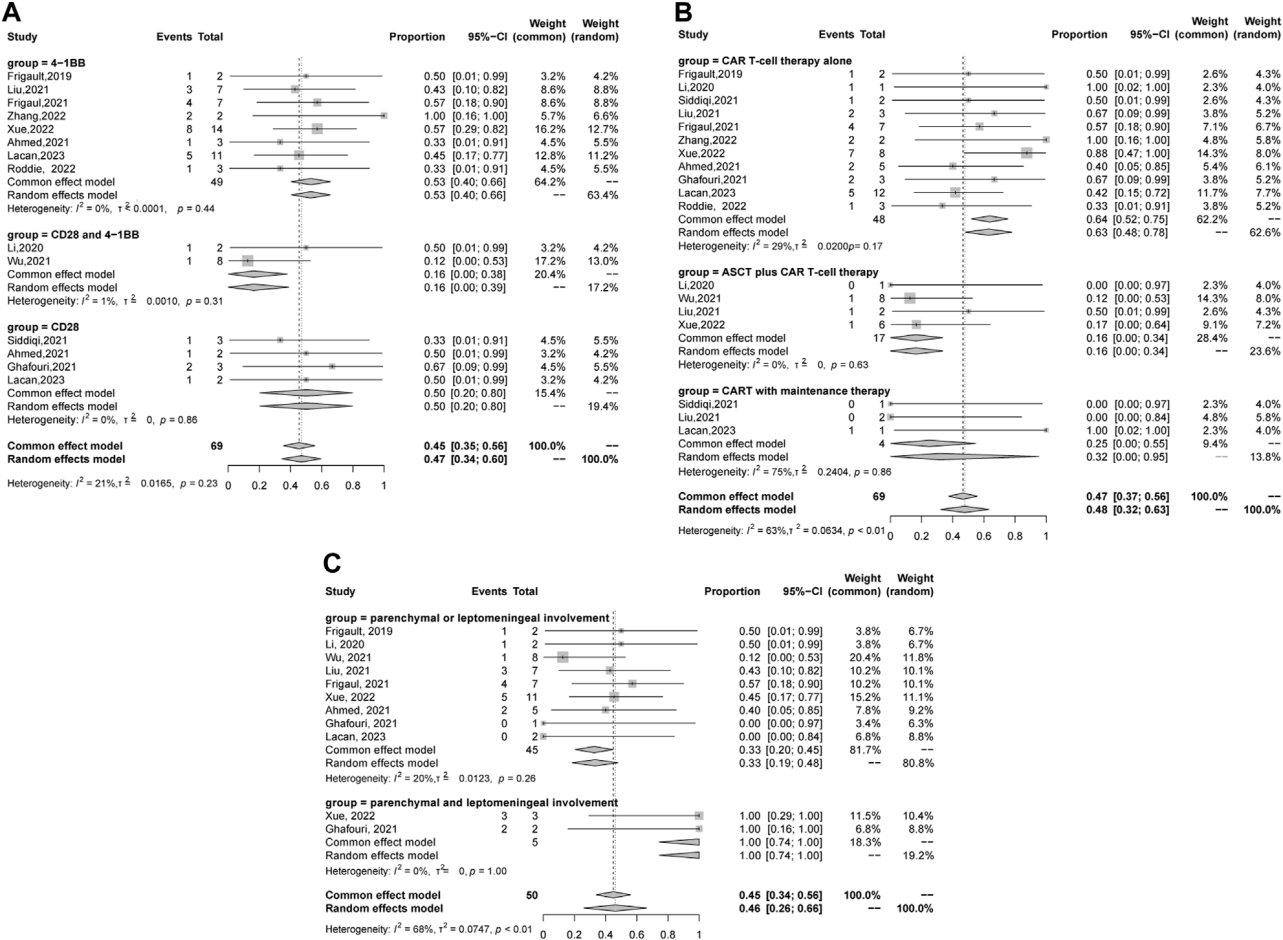
Forest plot of relapse rates and confidence intervals in CNSL patients. **(A)** Domain subgroup. **(B)** Therapy subgroup. **(C)** Sites of CNS involvement subgroup.

A total of 69 patients were evaluable for clinical response after BOR. The pooled relapse rate was 45% [95% CI 35, 56] (Figure 2). Subgroup analysis was performed, and the results are listed in Table 3. CAR T cells using the CD28/4-1BB domain had a lower relapse rate [16% (95% CI 0–38)] than those using the CD28 domain and 4-1BB domain [50% (95% CI 20–80) and 53% (95% CI 40–66), respectively] (*p* = 0.0151). Patients with parenchymal or leptomeningeal involvement had a lower relapse rate [33% (95% CI 19–48)] than those with both parenchymal and leptomeningeal involvement [100% (95% CI 74–100), *p* < 0.0001]. The relapse rate of patients was lower in the ASCT plus CAR T-cell therapy group [16% (95% CI 0–34)] than in the CAR T-cell with maintenance therapy group [32% (95% CI 0–95)] and CAR T-cell therapy alone group [64% (95% CI 52–75)] (*p* = 0.003) (Figure 3). Sex, age, CNS type, prior lines therapy, prior ASCT, status at CAR T-cell infusion, disease status, double hit rearrangement, and TP53 showed no correlation to the relapse rate.

**TABLE 3:** Subgroup analyses of relapse rates after BOR.

Prognostic factor	Events	n	I^2^ statistic	Relapse rate (95% CI)	Q	*p*
Overall	32	69	32%	0.45 [0.35; 0.56]		
Sex						
Female	15	33	42%	0.42 [0.27; 0.56]		
Male	8	21	0%	0.35 [0.17; 0.53]	0.30	0.5816
Age (years)						
≥60	7	18	34%	0.43 [0.17; 0.69]		
<60	18	39	0%	0.41[0.27; 0.56]	0.01	0.927
Domain						
CD28	5	10	0%	0.50 [0.20; 0.80]		
4-1BB	25	49	0%	0.53 [0.40; 0.66]		
CD28/4-1BB	2	10	1%	0.16[0.00; 0.38]	8.38	0.0151
CNS type						
PCNSL	11	27	51%	0.42 [0.19; 0.65]		
SCNSL	21	42	47%	0.50 [0.37; 0.63]	0.37	0.5412
Prior lines therapy						
≥5	8	15	60%	0.51 [0.35; 0.72]		
<5	21	49	0%	0.41 [028.; 0.54]	0.36	0.5489
Prior ASCT						
yes	10	19	26%	0.59 [0.40.; 0.78]		
no	17	38	51%	0.50 [0.30.; 0.69]	0.78	0.3775
Status at CART infusion						
SD or PD	25	44	59%	0.49 [0.30; 0.69]		
PR	3	16	78%	0.23 [0.00; 0.60]	1.47	0.2248
Disease status						
Isolated CNS	10	25	56%	0.46 [0.21; 0.70]		
Systemic + CNS	8	14	41%	0.56 [0.36; 0.75]	0.24	0.6242
Sites of CNS Involvement						
parenchymal or leptomeningeal involvement	17	45	20%	0.33 [0.19; 0.48]		
parenchymal and leptomeningeal involvement	5	5	0%	1.00 [0.74; 1.00]	21.58	<0.0001
Double hit rearrangement						
yes	1	3	-	-		
no	5	14	67%	0.31 [0.01; 0.62]	0.01	0.9673
TP53						
abnormal	4	7	88%	0.37 [0.14; 0.60]		
normal	6	15	0%	0.41 [0.00; 1.00]	0.01	0.9236
Therapy						
CAR-T alone	28	48	29%	0.64 [0.52; 0.75]		
ASCT plus CAR-T	3	17	0%	0.16 [0.00; 0.34]		
CAR-T with maintenance therapy	1	4	75%	0.32 [0.00; 0.95]	16.16	0.003

ASCT: autologous stem cell transplantation; CNS: central nervous system; CAR: chimeric antigen receptor; SCNSL: secondary central nervous system lymphoma; PCNSL: primary central nervous system lymphoma; PD: progressive disease; SD: stable disease; PR: partial remission; CR: complete response; NA: not available.PCNSL: primary central nervous system lymphoma; PD: progressive disease; SD: stable disease; PR: partial remission.

**FIGURE 4:**
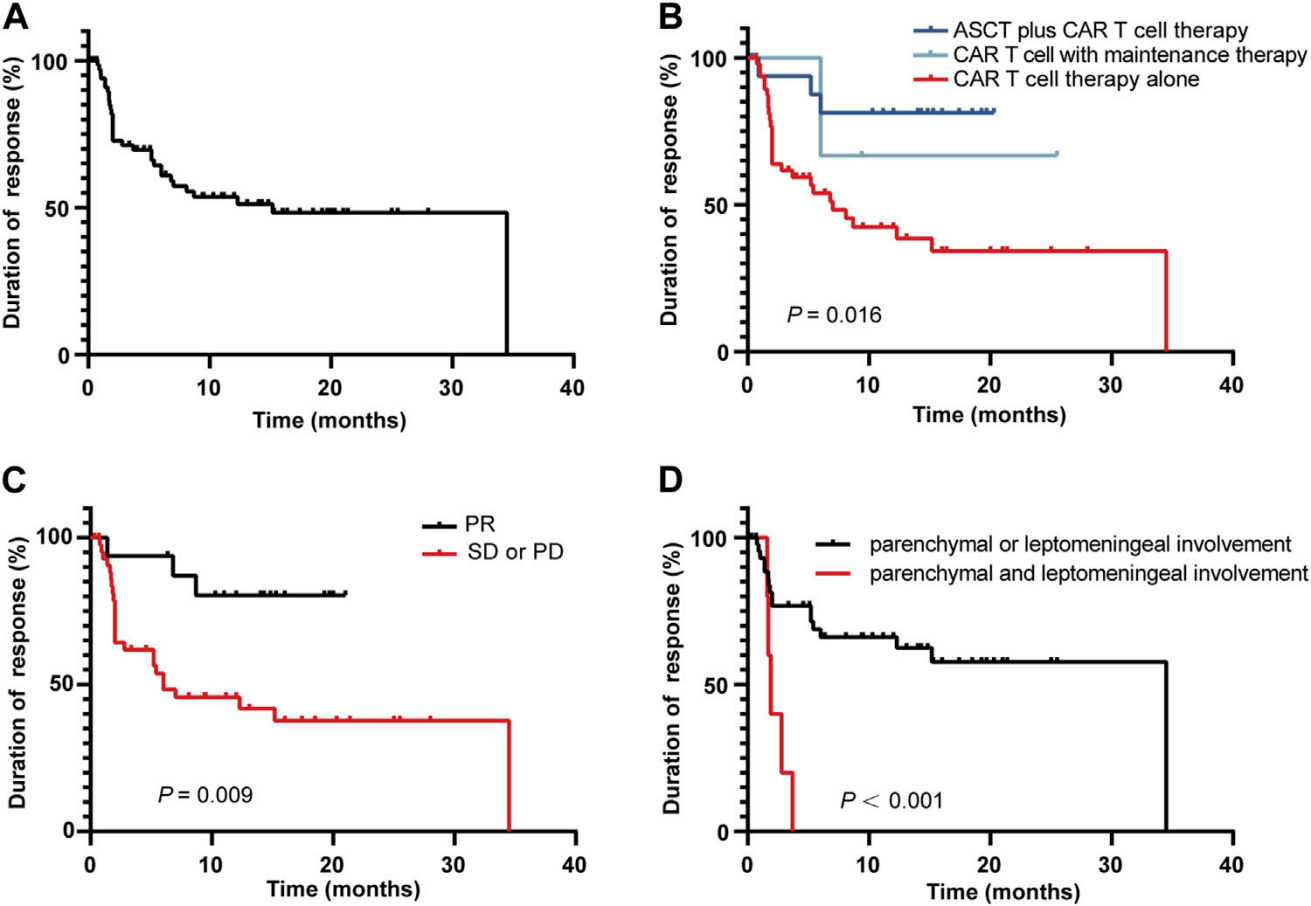
Kaplan-Meier curve of the DoR among the 69 responding patients **(A)**, in different therapy groups **(B)**, in patients with different disease statuses at CAR T-cell infusion **(C)**, and in different CNS involvement groups **(D)**.

The study showed that the median DoR was 9.9 months [95% CI 0.6, 27.3] Figure 4A. We next performed a subgroup analysis of DoR. Among the 69 enrolled patients, 17 patients received ASCT plus CAR T-cell therapy, 4 patients received CAR T-cell therapy with maintenance therapy, and the remaining 45 patients received CAR T-cell therapy alone. The longest DoR was observed in the ASCT plus CAR T-cell therapy group, followed by the CAR T-cell with maintenance therapy group, and the CAR T-cell alone therapy group had the shortest DoR (*p* = 0 .016) Figure 4B. The patients with a PR status at CAR T infusion had a longer DoR than patients with stable disease (SD) or progressive disease (PD) (*p* = 0 .009) Figure 4C. In addition, the DoR of patients with parenchymal or leptomeningeal involvement was superior to that of patients with parenchymal and leptomeningeal involvement (*p* < 0.001) Figure 4D.

**FIGURE 5:**
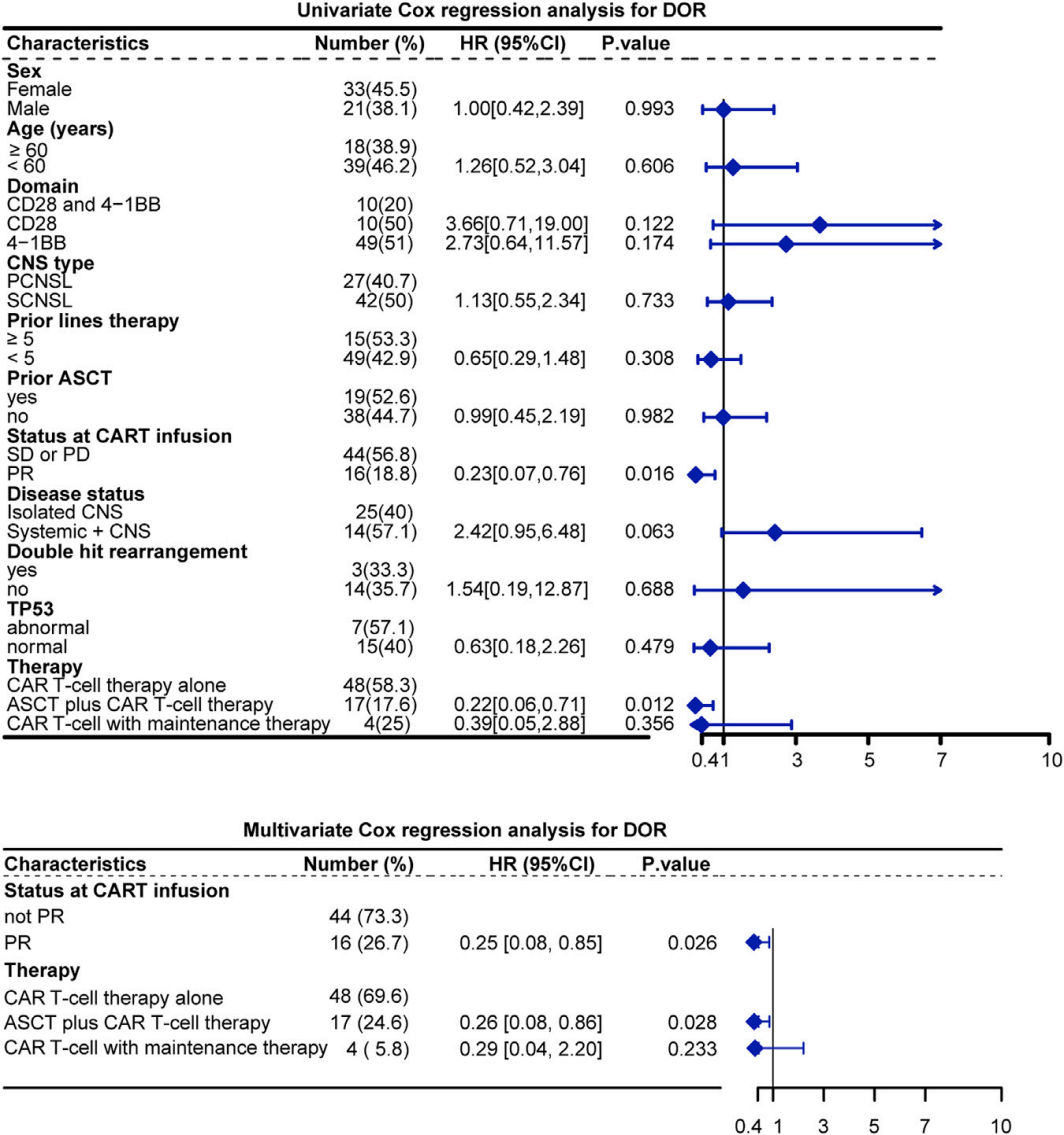
Univariate and multivariate analysis of DOR. Note: Sites of CNS involvement were excluded from Cox regression because the proportional hazards principle was not met.

To further confirm the effect of various variables on patient BOR, univariate and multivariate Cox proportional hazard analyses were performed to analyze the 12 identified studies included in the quantitative synthesis. From the multivariate analysis, the status at CAR T-cell infusion and therapy were significant risk factors for DOR. Specifically, the HR for relapse was 0.25 (*p* = 0.026) for the PR status at CAR T infusion and 0.26 (*p* = 0.028) for the ASCT plus CAR T-cell therapy Figure 5.

**FIGURE 6:**
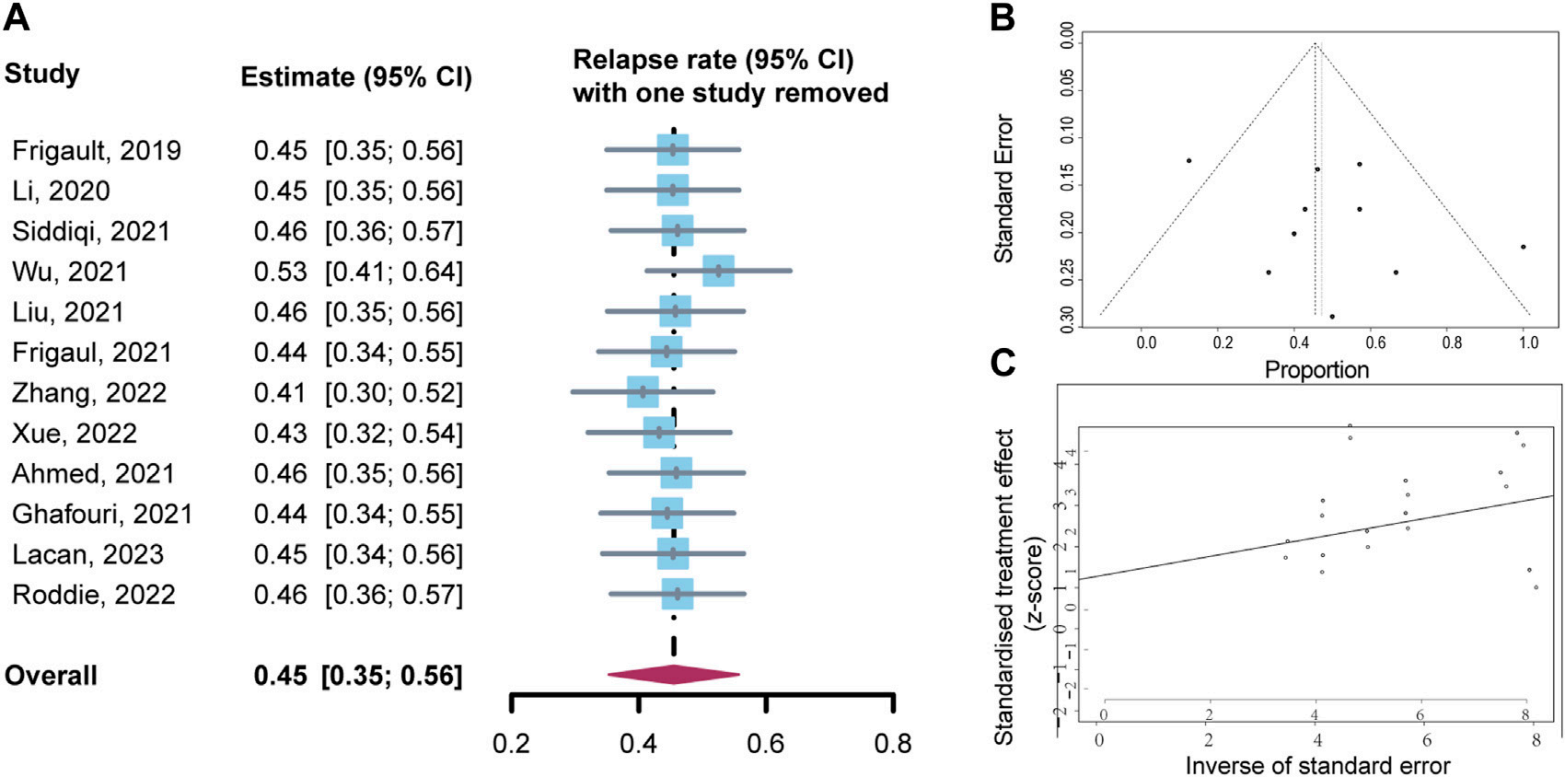
Sensitivity analysis and publication bias analysis for relapse rates. **(A)** Sensitivity analysis. **(B)** Funnel plot of publication bias. **(C)** Begg’s publication bias.

Sensitivity analysis was performed by evaluating the impact of excluding each study on the overall effect size Figure 6A. The results showed no significant change in the combined results, indicating that the study results were stable. The funnel plot revealed no indications of significant publication bias via visual inspection, which was further supported by Egger’s test and Begg’s test (*p* = 0.319 and 0.944, respectively) for publication bias Figure 6B, C.

## Discussion

The first-in-human clinical trial on CAR T-cell therapy for CNSL was reported by Abramson. A 68-year-old female, diagnosed as DLBCL with central nervous system involvement, experienced comprehensive remission of brain lesions, which verified the capability of CAR T-cells to breach the blood-brain barrier and elicit anti-tumor responses within the central nervous system (Abramson et al., 2017). Subsequently, the exclusion criteria of CAR T-cell therapy no longer categorize CNSL as an absolute contraindication. To confirm the sustained efficacy of CAR T-cell therapy in CNSL, we conducted a systematic review exclusively using studies that provided assessable individual data on DoR. The relapse rate of 69 CNSL patients enrolled in our study, who achieved an objective response (CR or PR) after CAR T-cell infusion, was 45%. This outcome aligns with the relapse rate observed in patients with relapsed/refractory B-cell malignancies undergoing CAR T-cell therapy, where 30%–50% of those who achieved CR experienced relapse, and most of them occurred within the initial year of treatment (Singh et al., 2020; Ghilardi et al., 2021; Xue et al., 2022). In this meta-analysis, we also noted that most relapse instances also occurred within 1 year.

CAR T-cell immunotherapy has presented a promising novel approach, and how to improve and prolong efficacy is still an urgent need. Our subgroup analysis revealed that the costimulatory domain of CAR T-cells can significantly influence sustained efficacy, with the lowest relapse rate in the CD28/4-1BB domain group. There was no obvious difference between the relapse rates of the CD28 and 4-1BB domain groups, which was different from previous reports on the prevalence of CAR T cells with 4-1BB than those with CD28 domains (Esensten et al., 2016; Salter et al., 2018). It is worth noting that the DoR of the CD28/4-1BB domain group presented a longer tendency compared to the CD28 and 4-1BB domain groups, but did not show a significant statistical difference in the univariate Cox regression model. Further research on a larger scale is required to determine whether CD28/4-1BB domain-engineered CAR T-cells enhance sustained efficacy.

Previous studies revealed that ASCT followed by CAR T-cell therapy showed a higher CR rate, better progression-free survival (PFS), and lower relapse/progression rate than ASCT therapy in relapsed or refractory DLBCL (Wang et al., 2022). Xue et al. reported that patients who received ASCT plus CAR T-cell therapy had significantly longer PFS and overall survival (OS) compared to those who received CAR T-cell therapy among 17 CNSL patients (Xue et al., 2022). Our findings offered further stronger confirmation of the aforementioned small-size study and indicated that CNSL patients who underwent ASCT in combination with CAR T-cell therapy had a longer DoR compared to those who received CAR T-cell therapy alone. Even after adjusting for multiple factors, this significant difference persisted. Anti-PD-1 therapy, lenalidomide, and ibrutinib have been proven to enhance the efficacy of CAR T-cell therapy (Chong et al., 2017; Thieblemont et al., 2020; Liu et al., 2021; Munoz et al., 2022; Sang et al., 2022). We noted that four CNSL patients received maintenance therapy subsequent to CAR T-cell therapy, including two with a PD1 inhibitor, one with lenalidomide, and one with ibrutinib. Patients who received CAR T-cell treatment combined with maintenance therapy appeared to exhibit an extended DoR compared with those who underwent CAR T-cell therapy alone; however, the trend was not statistically significant, likely attributed to the limited sample size and the lack of control in utilizing the same maintenance therapy.

We observed that the disease status at CAR T-cell infusion was associated with the sustained efficacy of treatment. Patients in PR at CAR T-cell infusion demonstrated a better DoR than patients in PD or SD, and the significant difference persisted even after adjusting for the received therapy. A meta-analysis of 38 reports containing 2,134 relapsed or refractory acute lymphoblastic leukemia patients revealed that pretreatment morphologic remission was associated with superior overall survival (Elsallab et al., 2023). No difference in DoR was observed between patients with prior therapy lines ≥5 and <5. These results indicated that selecting a suitable infusion time or implementing bridging therapy before treatment to manage the disease status is crucial for enhancing the effectiveness of CAR T-cell therapy. We also noted that patients with systemic disease and CNS disease presented a tendency of poorer DoR than patients with isolated disease. In addition, five patients with parenchymal and leptomeningeal involvement experienced total relapse after BoR, which was significantly higher than patients with parenchymal or leptomeningeal involvement in the DoR analysis. Studies have shown that patient age is associated with the efficacy of CAR T-cell treatment (Ghilardi et al., 2021). Our group analysis demonstrated that there was no difference in relapse rates between older patients (age ≥60 years) and younger patients (age <60 years). In four patients older than 75 years who achieved CR after CAR T-cell therapy in four included studies, only one experienced relapse in the follow-up period, while the longest DoR was 16 months. These results can provide advice on patient selection for CAR T-cell therapy in CNSL treatment. However, a larger scale prospective study is still needed to establish patient selection criteria, owing to the small number of patients.

Inevitably, there are some limitations in our systematic review and meta-analysis. First, due to the small number of relevant studies and cases, this meta-analysis included one conference abstract and some studies with a short follow-up time. Second, potential selection bias may exist, as prospective clinical studies and retrospective studies were enrolled at the same time. Third, we could not evaluate a wider range of effect modifiers among patients across different studies owing to a lack of patient-level data. Therefore, we look forward to more prospective studies with larger sample sizes in the future.

## Conclusion

In our study, we indicated the sustained efficacy of CAR T-cell therapy in CNSL based on individual data and analyzed the associated effect modifiers. The disease status at CAR T-cell infusion and combined treatment with CAR T-cell therapy had major impacts on the risk of relapse and DoR of patients. Further prospective large-scale studies are warranted to confirm the role of these effect modifiers in order to select the eligible population for and improve the sustained efficacy of CAR T-cell therapy in CNSL treatment.

## Data Availability

The raw data supporting the conclusion of this article will be made available by the authors, without undue reservation.

## Abbreviations

CNSL: Central nervous system lymphoma; CAR: Chimeric Antigen Receptor; DoR: Duration of Response; PCNSL: primary CNSL; SCNSL: secondary CNSL; DLBCL: Diffuse Large B-cell Lymphoma; PRISMA: Preferred Reporting Items for Systematic Reviews and Meta-Analyses; PROSPERO: Prospective Register of Systematic Reviews; MINORS: Methodological index for non-randomized studies; BOR: Best Overall Response; CI: Confidence interval; ASCT: Autologous stem cell transplantation; CNS: central nervous system; CR: complete response; PR: partial response; SD: stable disease; PD: progressive disease; PFS: progression-free survival; OS: overall survival.
